# Regulation of Syk activity by antiviral adaptor MAVS in FcεRI signaling pathway

**DOI:** 10.3389/falgy.2023.1098474

**Published:** 2023-04-24

**Authors:** Yuko Kawakami, Miho Kimura, Christella Widjaja, Kazumi Kasakura, Tomoaki Ando, Yu Kawakami, Joshua J. Obar, Toshiaki Kawakami

**Affiliations:** ^1^ Laboratory of Allergic Diseases, Center for Autoimmunity and Inflammation, La Jolla Institute for Immunology, La Jolla, CA, United States; ^2^ Department of Microbiology and Immunology, Geisel School of Medicine at Dartmouth College, Lebanon, NH, United States

**Keywords:** IgE, FcεRI, mast cells, RIG-I, MDA5, IRF3, antiviral signaling

## Abstract

**Background:**

Mast cells are the major effector cell type for IgE-mediated allergic reactions. Recent studies revealed a role for mast cells in orchestrating the host response to viral infections.

**Objective:**

We studied the relationship between FcεRI (high-affinity IgE receptor) and RIG-I-like receptor (RLR)-mediated antiviral signaling pathways.

**Methods:**

Mast cells (BMMCs) were cultured from bone marrow cells from mice deficient in MAVS or other RLR signaling molecules. MAVS expression was restored by retroviral transduction of MAVS-deficient BMMCs. These cells were stimulated with IgE and antigen and their activation (degranulation and cytokine production/secretion) was quantified. FcεRI-mediated signaling events such as protein phosphorylation and Ca^2+^ flux were analyzed by western blotting and enzyme assays. WT and mutant mice as well as mast cell-deficient *Kit^W−sh/W−sh^* mice engrafted with BMMCs were subjected to passive cutaneous anaphylaxis.

**Results:**

Unexpectedly, we found that mast cells devoid of the adaptor molecule MAVS exhibit dramatically increased cytokine production upon FcεRI stimulation, despite near-normal degranulation. Consistent with these observations, MAVS inhibited tyrosine phosphorylation, thus catalytic activity of Syk kinase, the key signaling molecule for FcεRI-mediated mast cell activation. By contrast, mast cells deficient in RIG-I, MDA5 or IRF3, which are antiviral receptor and signaling molecules upstream or downstream of MAVS, exhibited reduced or normal mast cell activation. MAVS-deficient mice showed enhanced late-phase responses in passive cutaneous anaphylaxis.

**Conclusion:**

This study demonstrates that the adaptor MAVS in the RLR innate immune pathway uniquely intersects with the adaptive immune FcεRI signaling pathway.

## Introduction

IgE plays a central role in the pathogenesis of asthma and allergic diseases (1, 2). FcεRI on mast cells consists of an IgE-binding α subunit, a signal-amplifying β subunit, and dimeric signal-triggering γ subunits. Binding of multivalent antigen (Ag) to FcεRI-bound IgE molecules crosslink FcεRI complexes; FcεRI crosslinking activates protein-tyrosine kinases (PTKs)-dependent signaling cascades, leading to the release of allergenic mediators (3). Among the several PTKs activated, Src family PTKs such as Lyn phosphorylate β and γ subunits of FcεRI, and Syk is recruited to tyrosine-phosphorylated γ subunits and phosphorylated at critical tyrosine residues (3, 4). Thus activated Syk plays a crucial role in most activation outcomes such as degranulation and cytokine production.

Mast cells are also implicated in virus infections (5) such as those with rhinovirus, respiratory syncytial virus and influenza virus. These respiratory viruses are the major pathogens associated with asthma exacerbations (6, 7). Mast cells orchestrate the host response to influenza A virus (8), as mast cell-deficient mice develop less influenza-associated morbidity (9). Furthermore, pre-seasonal treatment of asthmatic children with anti-IgE mAb omalizumab reduced frequencies of exacerbations induced by viral infections after a new school year started (10). Virus infection is detected by pattern recognition receptors such as Toll-like receptors (TLRs) and RIG-I-like receptors (RLRs). Recognition of viral RNA by RLRs (RIG-I and MDA5) leads to their interactions with MAVS (11–14). Activated MAVS adaptor complexes lead to activation of the transcription factors IRF3, IRF7, and NF-κB, resulting in production of type I interferons and inflammatory cytokines. Several studies showed synergistic interactions between TLRs and FcεRI signaling pathways to enhance the production of inflammatory cytokines (15, 16), whereas little is known about whether RLR pathways crosstalk with the FcεRI pathway. In this study, we studied the relationship between FcεRI and antiviral RLR signaling pathways. The BioGPS (http://biogps.org) database indicates that mouse mast cells express RLR signaling molecules such as RIG-I, MDA5, MAVS, and IRF3. Potential crosstalk between FcεRI and RLR signaling pathways was investigated initially using BMMCs derived from WT and *MAVS^−/−^* mice. Unexpected findings of increased production of cytokines in IgE/Ag-stimulated *MAVS^−/−^* BMMCs and enhanced PCA reactions in *MAVS^−/−^* mice were accounted for by increased Syk activity. Uniqueness of Syk regulation by MAVS was shown by analyzing mutant BMMCs lacking RIG-I, MDA5 or IRF3 molecules upstream or downstream of MAVS in RLR signaling pathways.

## Materials and methods

### Mice

*Cardif^−/−^* and WT mice in a C57BL/6 background originated from Jürg Tschopp's laboratory were donated by Sujan Shresta (La Jolla Institute for Immunology [LJI]) and renamed *MAVS^−/−^* mice to avoid confusions. These WT mice could have a substantial difference in the genetic background from other C57BL/6 mice. *Irf3^−/−^* mice were donated by Sonia Sharma (LJI). *C57BL/6-Kit^W−sh/W−sh^* mice were bred in house. Femurs from *Ddx58^−/−^* and *Ifh1^−/−^* mice were provided by Michael Gale, Jr. (University of Washington). Animal experiments were approved by the Animal Care and Use Committee of the LJI.

### Antibodies

Antibodies used in this study are listed in Table S1.

### Cultures of mast cells and retroviral transduction

Bone marrow cells were cultured in IL-3-containing medium, as described previously (17). Live cells were counted during weekly medium changes in the presence of Trypan Blue. Purity (>90%) of BMMCs was assessed by flow cytometry for FcεRI and c-Kit expression. Recombinant bicistronic retroviruses were generated by transfection of Plat-E cells with pMXpuro vector (18). BMMCs were infected with the retroviruses and selected by puromycin.

### Flow cytometry

Expression of c-Kit and FcεRI on mast cells was analyzed using FACSCalibur (BD Biosciences) after staining with APC-conjugated anti-c-Kit and FITC-conjugated IgE.

### Mast cell stimulation

BMMCs (2 × 10^6^ ml) were sensitized overnight with 0.5 μg/ml anti-DNP IgE. Cells washed with and resuspended in Tyrode buffer (2 × 10^6^ ml) were stimulated with DNP_23_-HSA for 45 min. The amount of β-hexosaminidase in supernatants was measured using *p*-nitrophenyl N-acetyl β-D-glucosaminide as substrate and spectrophotometer (at 405 nm). Supernatants of IgE-sensitized BMMCs stimulated with Ag for 20 h were measured by ELISA kits for IL-2, IL-6, TNF (BD Biosciences) and IL-13 (eBiosciences).

### Ca^2+^ flux

IgE-sensitized BMMCs were loaded with Indo 1-AM (Calbiochem) and stimulated with Ag or anti-IgE at the indicated concentrations as previously described, except that the fluorescence ratio was continuously monitored using a BD-LSR II flow cytometer.

### Mast cell engraftment

BMMCs derived from WT and MAVS^−/−^ mice were transferred by intravenous injection (5 × 10^6^ cells in 200 μl PBS) into 4-week-old female *Kit^W−sh/W−sh^* mice and used 8 weeks later. Proper engraftment of transferred mast cells in the ears was confirmed by staining by toluidine blue.

### Immunoblotting

Mast cells appropriately stimulated were lysed in 1% NP-40 lysis buffer. Lysates or immunoprecipitates were analyzed by SDS-PAGE followed by electroblotting to PVDF membranes (PerkinElmer). Membranes were incubated with a primary antibody and then with an HRP-conjugated secondary antibody. Antibody-bound proteins were revealed by ECL reagent (PerkinElmer).

### PCA experiments

Mice were sensitized by intradermal injection of IgE into the ear with 0.5 μg of anti-DNP IgE mAb. 24 h late, 10 μl 1-fluoro-2,4-dinitrobenzene (0.3%) in acetone/olive oil (4:1) was applied onto both sides of an ear. For control, the ear is applied with 10 μl acetone/olive oil solution. Ear thickness was measured at 0.5–48 h after challenge using a caliper (Mitutoyo).

### Statistical analysis

Statistical analysis was performed with two-tailed Student's *t*-test using Prism software (Graphpad). *P* < 0.05 was considered statistically significant.

## Results and discussion

### Increased production/secretion of cytokines by FcεRi-stimulated *mavs^−/−^* mast cells

**Figure 1:**
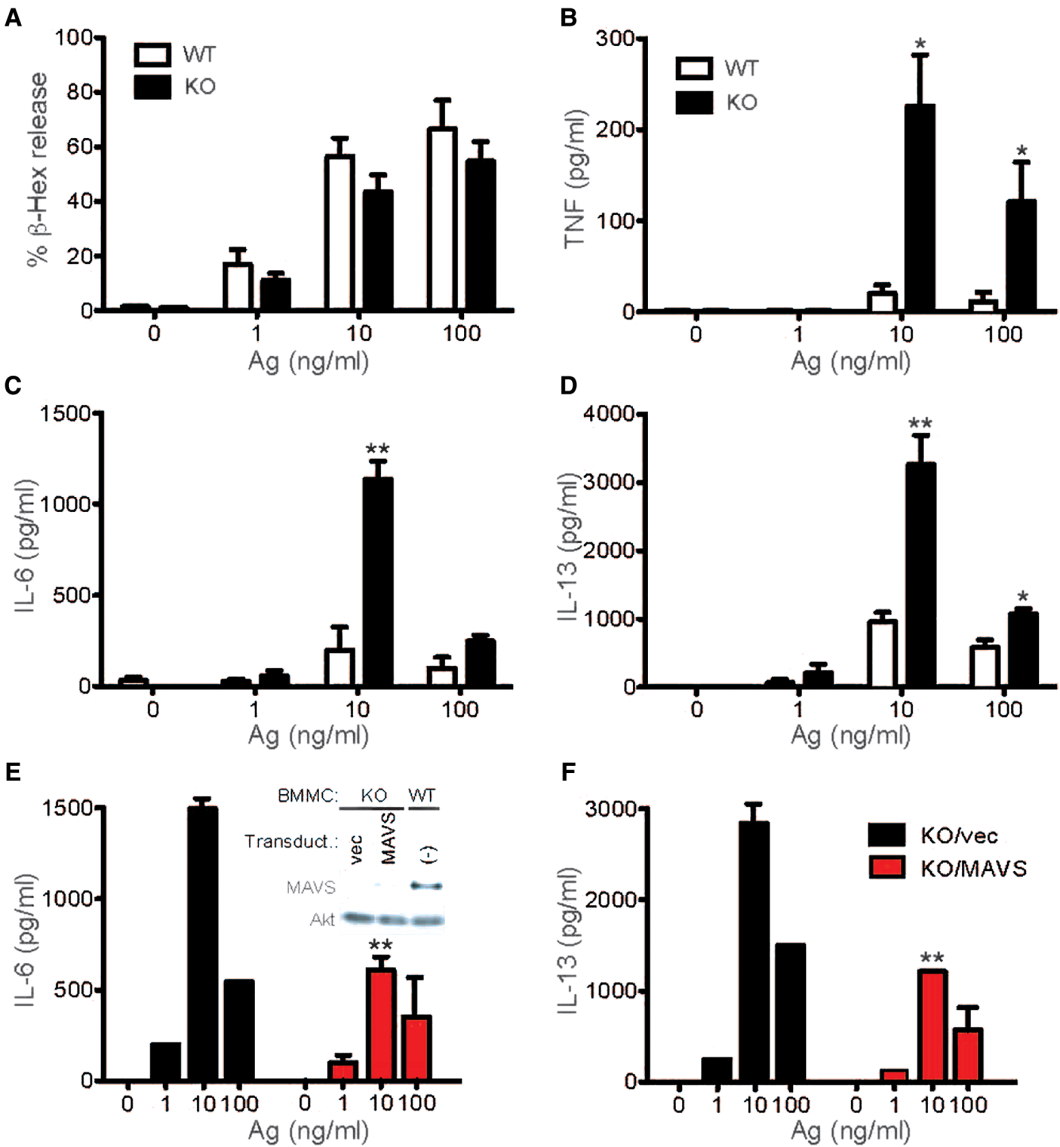
MAVS-deficient mast cells secrete increased cytokines upon FcεRI stimulation. (**A–D**) WT and *MAVS^−/−^* BMMCs were sensitized with anti-DNP IgE and stimulated with the indicated concentrations of DNP_23_-HSA (Ag) for 45 min (for β-hexosaminidase release) or 20 h (for secretion of cytokines). More than 5 independent experiments on Syk phosphorylation were performed by 3 different researchers with similar results. Ratios of phosphorylated Syk to Syk amount were calculated by densitometry (**A, Right**). (**E,F**) *MAVS^−/−^* BMMCs were infected with bicistronic retrovirus encoding MAVS or empty vector (vec). Puromycin-resistant cells were subjected to IgE sensitization and Ag stimulation. Inset in panel E shows expression of MAVS in *MAVS^−/−^* BMMCs and WT BMMCs along with Akt expression in these cells. Results from 3 transduction experiments are shown. Mean ± SEM are shown. *, **: *p* < 0.05, *p* < 0.01 vs. WT cells (**B-D**) or empty vector-transduced cells (**E,F**) by Student's *t*-test.

Since MAVS plays an essential function in RLR signaling and leads to the production of inflammatory cytokines, many of which are also produced by activated mast cells, we tested whether MAVS affects FcεRI signaling. Mast cells (BMMCs) developed normally from bone marrow cells of *Mavs^−/−^* mice in IL-3-containing culture medium and proliferated similarly to stem cell factor, the crucial mast cell growth factor (Supplementary Figure S1A,B). Cell deaths induced by IL-3 deprivation were also normal (Supplementary Figure S1C). Thus, MAVS was dispensable for the differentiation, proliferation, and survival of BMMCs. When stimulated with IgE and Ag, *Mavs^−/−^* BMMCs degranulated almost normally (Figure 1A). However, production of TNF, IL-6 and IL-13 was dramatically increased in *Mavs^−/−^* BMMCs, compared with WT cells (Figure 1B-D). The increased cytokine production in *Mavs^−/−^* BMMCs was due to the lack of MAVS because restoration of MAVS expression in *Mavs^−/−^* mast cells by retroviral transduction reduced cytokine production close to WT levels (Figure 1E,F). These results indicate that there is a molecular crosstalk between the two signaling pathways for innate antiviral and FcεRI-mediated adaptive immune responses.

### Increased tyrosine phosphorylation of Syk in *mavs^−/−^* mast cells

**Figure 2:**
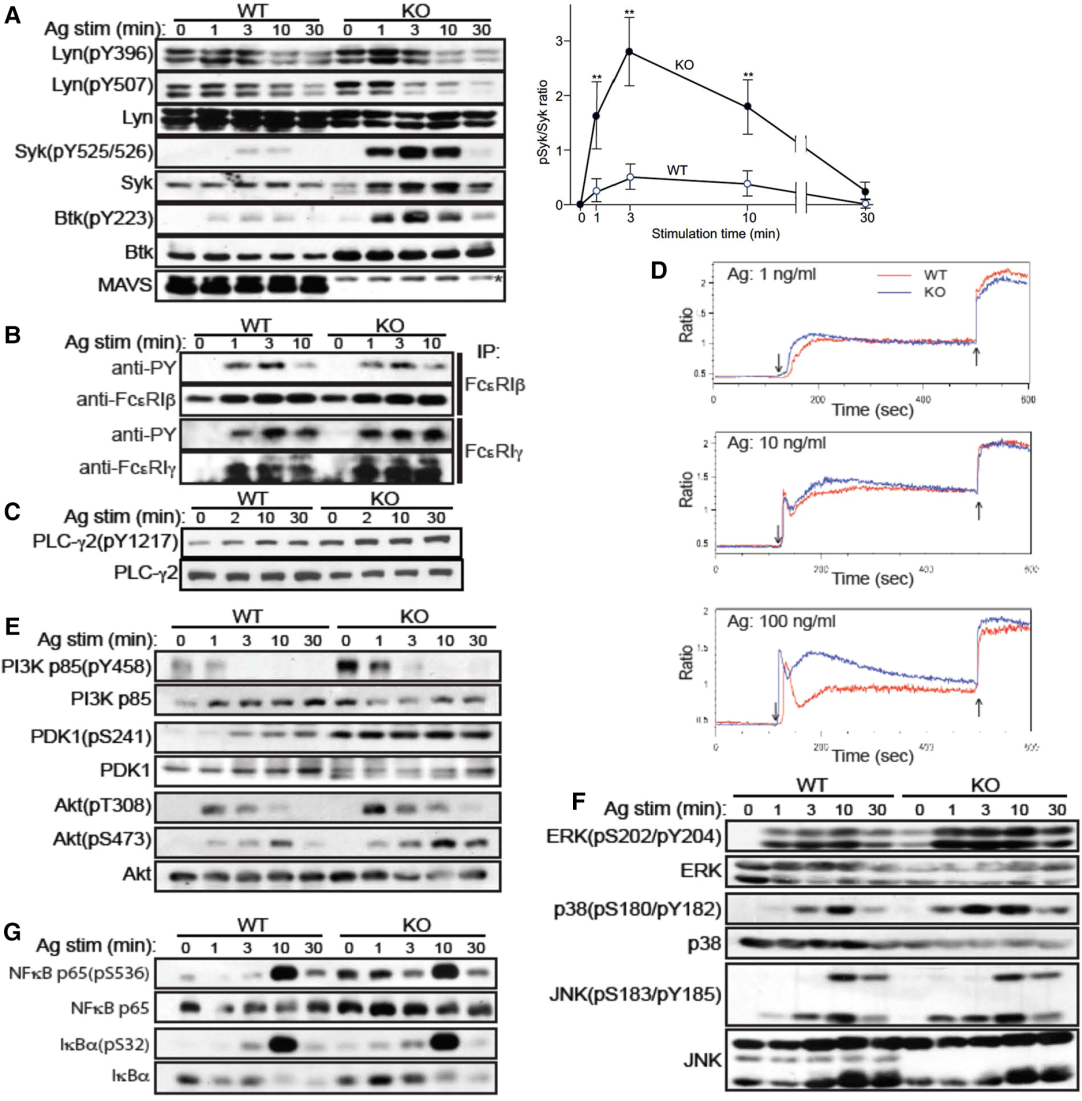
FcεRI-stimulated MAVS-deficient mast cells exhibit dramatically enhanced Syk activation. (**A-C, E-G**) WT and *MAVS^−/−^* BMMCs were sensitized with IgE and stimulated with Ag (100 ng/ml of DNP_23_-HSA). Cells were lysed, and cleared lysates were either directly analyzed by SDS-PAGE followed by western blotting (**A,C,E-G**) or first immunoprecipitated (IP) before the precipitates were analyzed by SDS-PAGE (**B**). Proteins detected by antibody are indicated on the left of the gels. (**D**) IgE-sensitized BMMCs were loaded with Indo-AM and stimulated with Ag (↓), followed by ionomycin (↑). Fluorescence ratio (525:405 nm) was measured by flow cytometer. Results representative of two independent experiments are shown except for Syk phosphorylation, which was confirmed by two additional experiments.

In order to gain mechanistic insights into increased cytokine production in *Mavs^−/−^*BMMCs, we compared FcεRI signaling events (Supplementary Figure S2) between WT and *Mavs^−/−^* BMMCs. Phosphorylations of Lyn, one of the earliest activated PTKs (19), at Tyr-396 in the activation loop and at Tyr-507 in the negative regulatory site were comparable in WT and *Mavs^−/−^* BMMCs (Figure 2A). As Lyn phosphorylates β and γ subunits of FcεRI (19, 20), tyrosine phosphorylation of the β and γ subunits of FcεRI was similar in *Mavs^−/−^* and WT cells (Figure 2B). Although we did not know why, we noticed that anti-β and particularly anti-γ antibodies immunoprecipitate tyrosine-phosphorylated subunits better than non-tyrosine-phosphorylated subunits. Lyn expression was lower in *Mavs^−/−^*than in WT cells (Figure 2A). However, phosphorylation of FcεRI β and γ subunits was not affected by Lyn expression levels. Remarkably, phosphorylation of Syk at its activation loop, which is essential for Syk function (21), was dramatically increased in Ag-stimulated *Mavs^−/−^* BMMCs (Figure 2A), indicating that Syk activity is much higher in *Mavs^−/−^* cells. Indeed, several known Syk phosphorylation targets, e.g., Btk, phospholipase C (PLC)-γ2 and p85 subunit of phosphatidylinositol 3-kinase (PI3K), were more highly phosphorylated in *Mavs^−/−^* than in WT cells (Figures 2A,C,E). Furthermore, Syk activity might be higher even before Ag stimulation: phosphorylation of direct Syk targets (Btk, PLC-γ2, p85 PI3K) and downstream signaling molecules (PDK1, NF-κB p65, ERK1/2) was increased before Ag stimulation. Consistent with the increased tyrosine phosphorylation of PLC-γ2, Ca^2+^ responses were enhanced in IgE/Ag-stimulated *Mavs^−/−^* mast cells (Figure 2D). Expression levels of Syk, Btk and PLC-γ2 were also affected by MAVS deficiency (Figure 2A,C).

Cytokine production in mast cells are controlled by Akt (22), MAPK (23), and NF-κB (22). Akt activates several transcription factors including AP-1 and NF-AT (22). Akt activity is regulated by phosphorylation at Thr-308 and Ser-473 by PDK1 and PKC-βII, respectively, in FcεRI-stimulated mast cells (24, 25). PDK1 is activated by PI3K products (24). As shown in Figure 2E, PI3K p85 was highly phosphorylated at baseline in *Mavs^−/−^* cells, indicating higher PI3K activity. PDK1 phosphorylation is constitutively higher in *Mavs^−/−^* than WT cells, as was Ag-induced Akt phosphorylation at both Thr-308 and Ser-473 in *Mavs^−/−^* cells (Figure 2E). Phosphorylation of MAPKs, i.e., ERK1, ERK2, and p38, was increased in *MAVS^−/−^*cells, whereas that of JNK1 and JNK2 was comparable in the two cell types (Figure 2F). Thus, increased activity of ERK1, ERK2, and p38 likely contributes to the increased cytokine production in *Mavs^−/−^*mast cells. The activity of another transcription factor NF-κB also seemed to be slightly increased in *Mavs^−/−^* cells, as phosphorylation of IκBα and p65 was increased in *Mavs^−/−^* cells (Figure 2G). Altogether, Akt, ERK1/2, p38, and NF-κB likely contributed to the increased cytokine production in *Mavs^−/−^* cells.

### Enhanced late-phase and chronic anaphylactic responses in *mavs^−/−^* mice

**Figure 3:**
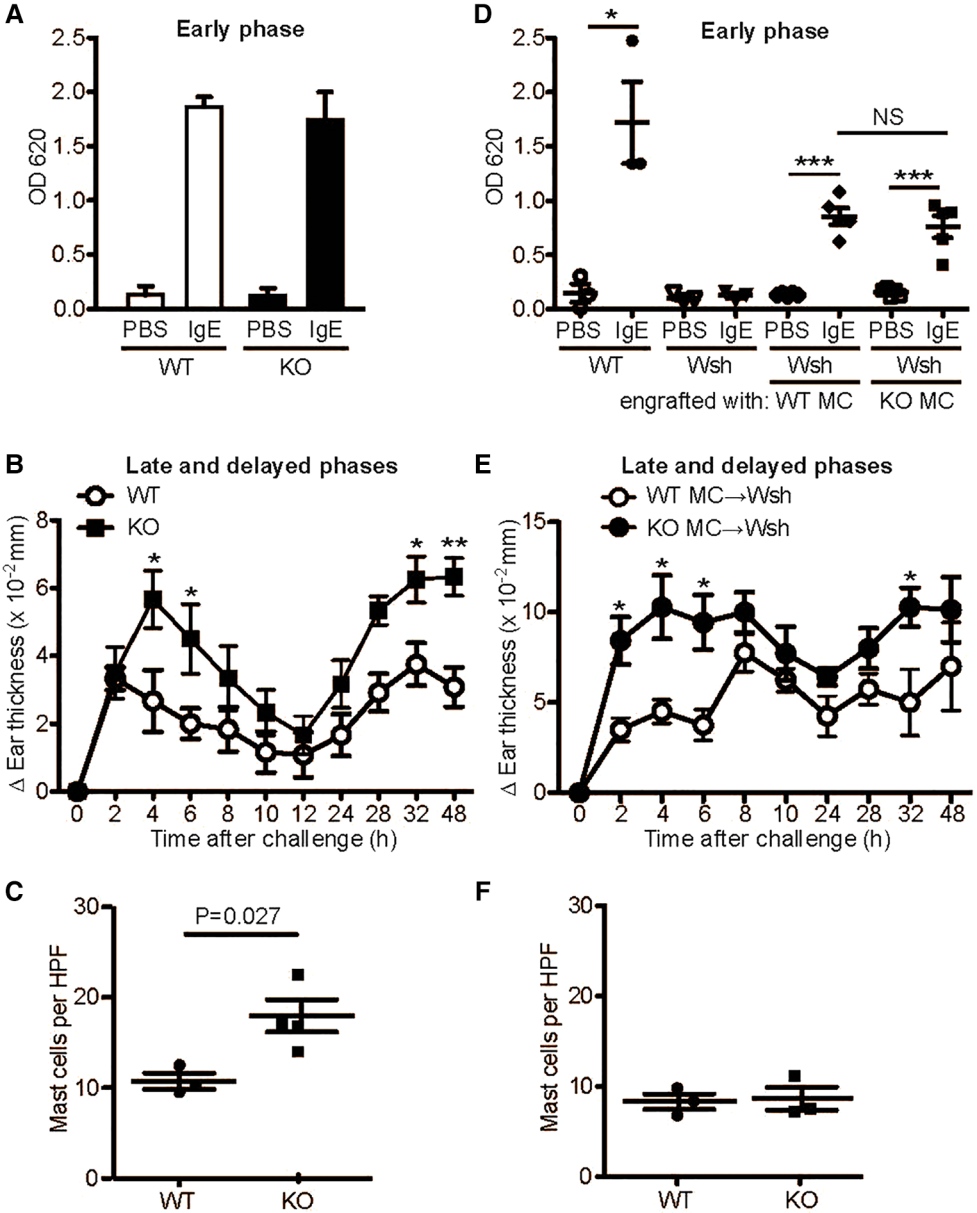
MAVS-deficient mice exhibit enhanced late and delayed, but normal acute, PCA responses. 6−8 week-old WT and *MAVS^−/−^* mice (**A-C**) or *Kit^W−sh/W−sh^* (Wsh) mice engrafted with WT or *MAVS^−/−^* BMMCs (**D-F**) were sensitized by administration of 500 ng anti-DNP IgE or PBS (10 μl) to each ear. 24 h later, mice were challenged intravenously with 100 μg DNP_23_-HSA in 100 μl 1% Evans blue (**A,D**). Evans blue was extracted from the ears 30 min after Ag injection. Alternatively, 10 μl of 0.3% dinitrofluorobenzene in acetone and olive oil (4:1) was painted on both sides of ears (**B,E**). Ear thickness was measured. Results representative of three independent experiments using 4−6 mice per group are shown. *, **, ***: *p* < 0.05, *p* < 0.01, *p* < 0.001 by Student's *t*-test. NS, not significant. (**C,F**) Mast cells in the ear of naïve WT and *MAVS^−/−^* mice (**C**) and mast cell-engrafted *Kit^W−sh/W−sh^* mice (**F**). Mast cells in the ears were stained by toluidine blue. HPF, high power field.

We next examined the effect of MAVS deficiency on mast cell-dependent *in vivo* allergic reactions, i.e., passive cutaneous anaphylaxis (PCA). Ag stimulation of IgE-sensitized mice induced early and late-phase reactions. Consistent with the comparable degranulation in WT and *MAVS^−/−^*BMMCs, early PCA responses, which are mainly due to histamine released from activated mast cells (26), were similar between WT and *Mavs^−/−^* mice (Figure 3A). By contrast, late-phase responses at 4–6 h after Ag stimulation, which are dependent partly on TNF secreted from activated mast cells (27), as well as the more chronic phase of 32–48 h were higher in *Mavs^−/−^* than in WT mice (Figure 3B). Concerned with increased mast cells in the ear of naïve *Mavs^−/−^* mice (Figure 3C), we wished to compare the contributions of WT vs. *Mavs^−/−^* mast cells to PCA under conditions with similar numbers of mast cells. To this end, we first engrafted WT or *Mavs^−/−^* BMMCs into mast cell-deficient *Kit^W−sh/W−sh^* mice. After the presence of comparable numbers of mast cells in the ears was confirmed 8 weeks after the engraftment (Figure 3F), the mice were subjected to PCA experiments. *Kit^W−sh/W−sh^* mice engrafted with *Mavs^−/−^* BMMCs exhibited stronger late-phase and chronic-phase responses than in *Kit^W−sh/W−sh^* mice engrafted with WT BMMCs (Figure 3E), whereas early responses were comparable in the two groups (Figure 3D). Therefore, these *in vivo* results faithfully reflect the higher-than-WT TNF-producing phenotypes of *Mavs^−/−^* mast cells.

### RIG-I, but not MDA5 or IRF3, is required for cytokine production in IgE/Ag-stimulated mast cells

**Figure 4:**
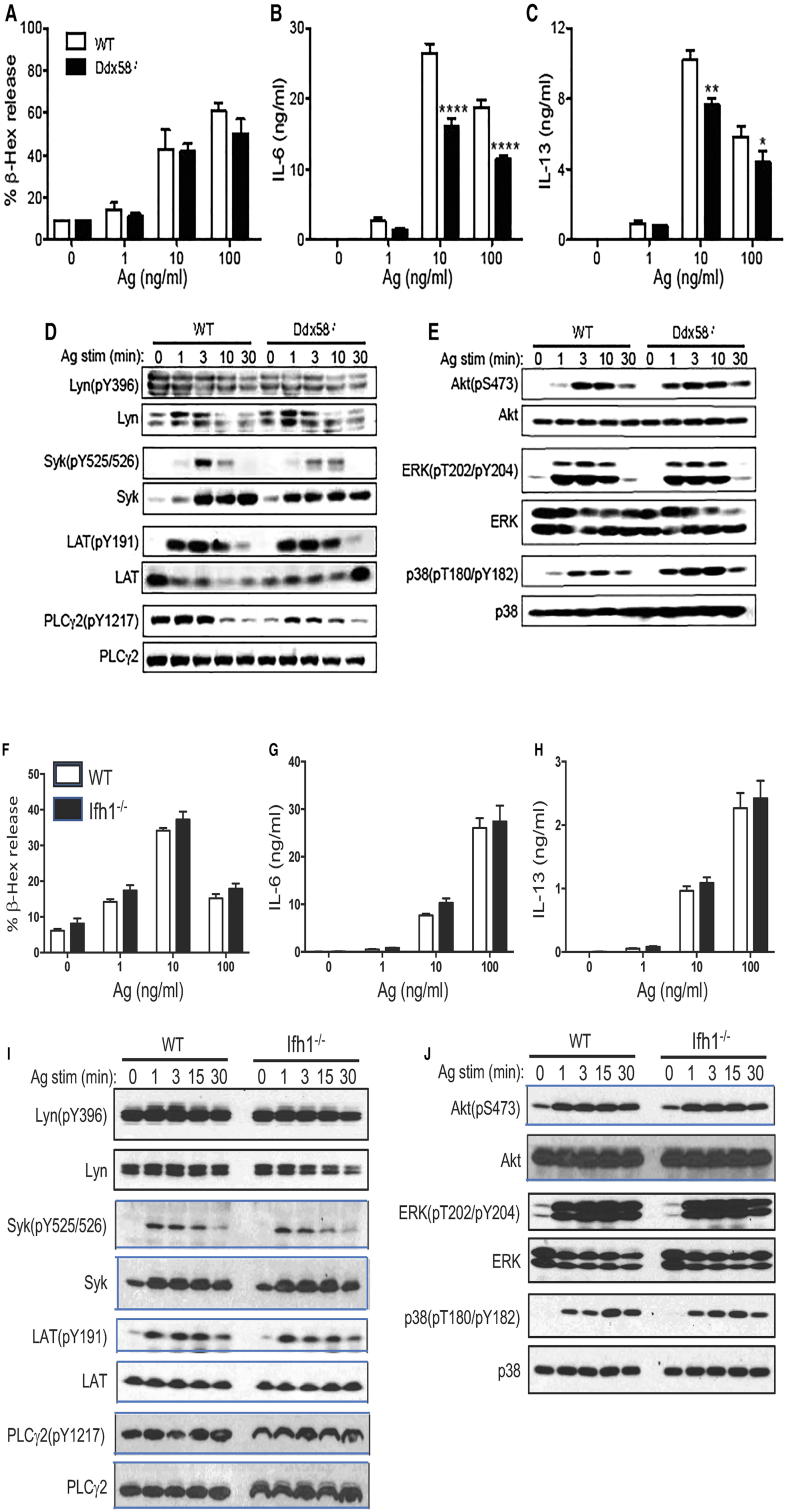
FcεRI-stimulated biologic and signaling outcomes in *Ddx58^−/−^*, *Ifh1^−/−^* and *Irf3^−/−^* BMMCs. WT, *Ddx58^−/−^* (**A-E**), *Ifh1^−/−^* (**F-J**) and *Irf3^−/−^* (**K-O**) BMMCs were sensitized with anti-DNP IgE and stimulated with the indicated concentrations of DNP_23_-HSA (Ag) for 45 min (for degranulation) or 20 h (for secretion of cytokines). Results representative of two independent experiments are shown. Signaling analysis was conducted once as described in Figure 2 legend.

An interesting question arising from the above results is whether MAVS-mediated inhibition of cytokine production in IgE/Ag-stimulated mast cells is simply due to Syk inhibition by MAVS or through the effect on the RIG-I/MAVS pathway (Supplementary Figure S2). To distinguish between these possibilities, we studied BMMCs derived from mice deficient in RIG-I, MDA5 or IRF3, two viral RNA sensors upstream of or the transcription factor downstream of MAVS, respectively. *Ddx58^−/−^* (RIG-I-deficient) and *Ifh1^−/−^* (MDA5-deficient) BMMCs developed normally. While RIG-I-deficient cells degranulated normally upon FcεRI stimulation (Figure 4A), cytokine production from RIG-I-deficient BMMCs was reduced by 20%–40% (Figure 4B,C), in sharp contrast with *Mavs^−/−^* BMMCs. Consistent with these results, Syk phosphorylation was slightly reduced, whereas Lyn phosphorylation at Tyr-396 was comparable in WT and RIG-I-deficient BMMCs (Figure 4D). Phosphorylation of PLC-γ2 was reduced in RIG-I-deficient BMMCs at early time points (Figure 4D). Phosphorylation of Akt and ERK1/2 was similar between WT and RIG-I-deficient BMMCs while phosphorylation of p38 was increased in RIG-I-deficient BMMCs (Figure 4E). On the other hand, degranulation and cytokine production were normal in MDA5-deficient cells (Figure 4F-H). In line with this activation phenotype, the phosphorylation events including Lyn, Syk, LAT, PLC-γ2, Akt, ERK1/2, and p38 were all indistinguishable between WT and MDA5-deficient BMMCs (Figure 4I-J). Unlike *Mavs^−/−^* BMMCs, expression of Lyn and Syk was comparable between RIG-I-deficient, MDA5-deficient, and WT BMMCs. Some BMMCs produce ten times the amount of IL-13 (Figure 4C) than in the other cells. We think that the differences in cytokine production could be due to differences in the genetic background of the mice used.

**Figure:**
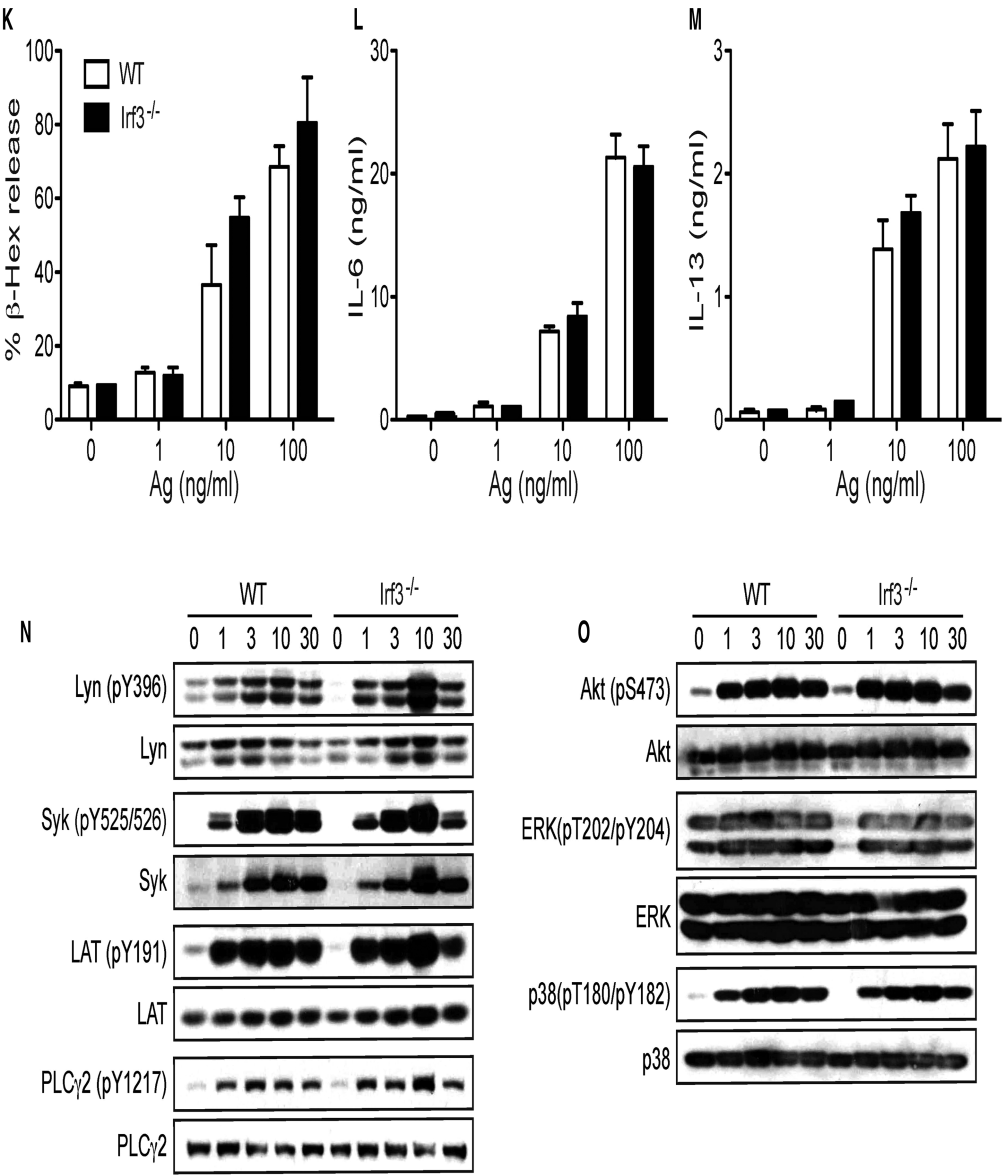


*Irf3^−/−^* BMMCs also developed normally. These cells degranulated normally (Figure 4K) and produced cytokines at WT levels upon FcεRI stimulation (Figure 4L,M). The early signaling events in IgE/Ag-stimulated *Irf3^−/−^* BMMCs were similar to WT cells, except for increased phosphorylation of Lyn (pY396), Syk, LAT (pY191) and PLC-γ2 (pY1217) at 10 min stimulation (Figure 4N). Phosphorylation of Akt, ERK1/2, and p38 were comparable in the two cell types (Figure 4O).

In summary, this study suggests that the adaptor protein MAVS of the antiviral RLR signaling pathway intersects with the FcεRI-mediated activation pathway, by inhibiting the key signaling molecule Syk and/or increased expression of Btk (18) and other signaling proteins. The inhibitory function of MAVS in FcεRI signaling seems unique among the signaling molecules involved in the RLR-mediated innate antiviral pathway. RIG-I plays a minor positive regulatory role in FcεRI signaling, but MDA5 and IRF3 do not influence FcεRI signaling. Differences in potential effects on FcεRI signaling between RIG-I and MDA5 might be translated to effects of viral infections on FcεRI signaling. For example, activation of RIG-I vs. MDA5 by different respiratory viruses might have different qualitative and quantitative effects on asthma exacerbation (28). Our observations on unique effects of RLR signaling molecules on the FcεRI pathway imply that the FcεRI signaling pathway evolved by utilizing existent innate immune signaling molecules in a unique, non-systematic way.

## Data Availability

The original contributions presented in the study are included in the article/**Supplementary Materials**, further inquiries can be directed to the corresponding author.
