# Supplementary material for: Regulation of Syk activity by antiviral adaptor MAVS in FcεRI signaling pathway
Source: Front Allergy. 2023 Apr 24;4:1098474. doi: 10.3389/falgy.2023.1098474 (PMC10165108; doi:10.3389/falgy.2023.1098474)
Supplement: Supplementary file 1 [file Datasheet1.pdf]

Supplementary Material

Table S1. Antibodies used in this study

**Antigen**	**clone**	**conjugated with**	**Provider**
c-Kit	Rat mAb 2B8	APC-Cy7	BioLegend
FcεRIα	Hamster mAb MAR-1	PE	BioLegend
FcεRIβ	Rat mAb JRK	unconjugated	Juan Rivera, NIH
FcεRIγ	Rabbit pAb	unconjugated	ThermoFisher
MAVS	Rabbit pAb	unconjugated	Cell Signaling Technology
Lyn	Rabbit pAb 44	unconjugated	Santa Cruz Biotechnology
pSrc(Lyn pY396)	Rabbit pAb	unconjugated	Cell Signaling Technology
pLyn(pY507)	Rabbit pAb	unconjugated	Cell Signaling Technology
Syk	Rabbit mAb D3Z1E	unconjugated	Cell Signaling Technology
Syk(pY525/526)	Rabbit mAb C87C1	unconjugated	Cell Signaling Technology
Btk	Rabbit pAb D3H5	unconjugated	Cell Signaling Technology
Btk(pY223)	Rabbit mAb D9T6H	unconjugated	Cell Signaling Technology
pY(phosphotyrosine)	Mouse mAb 4G10	unconjugated	Upstate Biotechnologies
PLC-γ2	Rabbit pAb	unconjugated	Cell Signaling Technology
PLC-γ2(pY1217)	Rabbit pAb	unconjugated	Cell Signaling Technology

PI3K p85	Rabbit pAb	unconjugated	Cell Signaling Technology
PI3K p85(pY458)	Rabbit mAb E3U1H	unconjugated	Cell Signaling Technology
PDK1	Rabbit pAb	unconjugated	Cell Signaling Technology
PDK1 (pS241)	Rabbit mAb C49H2	unconjugated	Cell Signaling Technology
Akt (pan)	Rabbit mAb C67E7	unconjugated	Cell Signaling Technology
Akt (pT308)	Rabbit mAb D25E6	unconjugated	Cell Signaling Technology
Akt (pS473)	Rabbit mAb D9E	unconjugated	Cell Signaling Technology
ERK1/2	Rabbit mAb 137F5	unconjugated	Cell Signaling Technology
ERK (pS202/pY204)	Rabbit mAb D13.14.4E	unconjugated	Cell Signaling Technology
JNK1/2	Rabbit pAb	unconjugated	Cell Signaling Technology
JNK(pS183/pY185)	Rabbit pAb	unconjugated	Cell Signaling Technology
p38α (C-20)	Rabbit pAb	unconjugated	Santa Cruz Biotechnology
p38(pS180/pY182)	Rabbit pAb	unconjugated	Cell Signaling Technology
NF-κB p65	Rabbit mAb D14E12	unconjugated	Cell Signaling Technology
NF-κB p65(pS536)	Rabbit mAb 93H1	unconjugated	Cell Signaling Technology
IκBα (C-21)	Rabbit pAb	unconjugated	Santa Cruz Biotechnology
IκBα(pS32)	Rabbit mAb 14D4	unconjugated	Cell Signaling Technology
LAT	Rabbit pAb	unconjugated	Cell Signaling Technology
LAT(pY191)	Rabbit pAb	unconjugated	ThermoFisher
RIG-I	D14G6	unconjugated	Cell Signaling Technology

MDA5	Rabbit mAb D74E4	unconjugated	Cell Signaling Technology
IRF3	Rabbit mAb EPR2418Y	unconjugated	Abcam
IRF7	Rabbit mAb D2A1J	unconjugated	Cell Signaling Technology

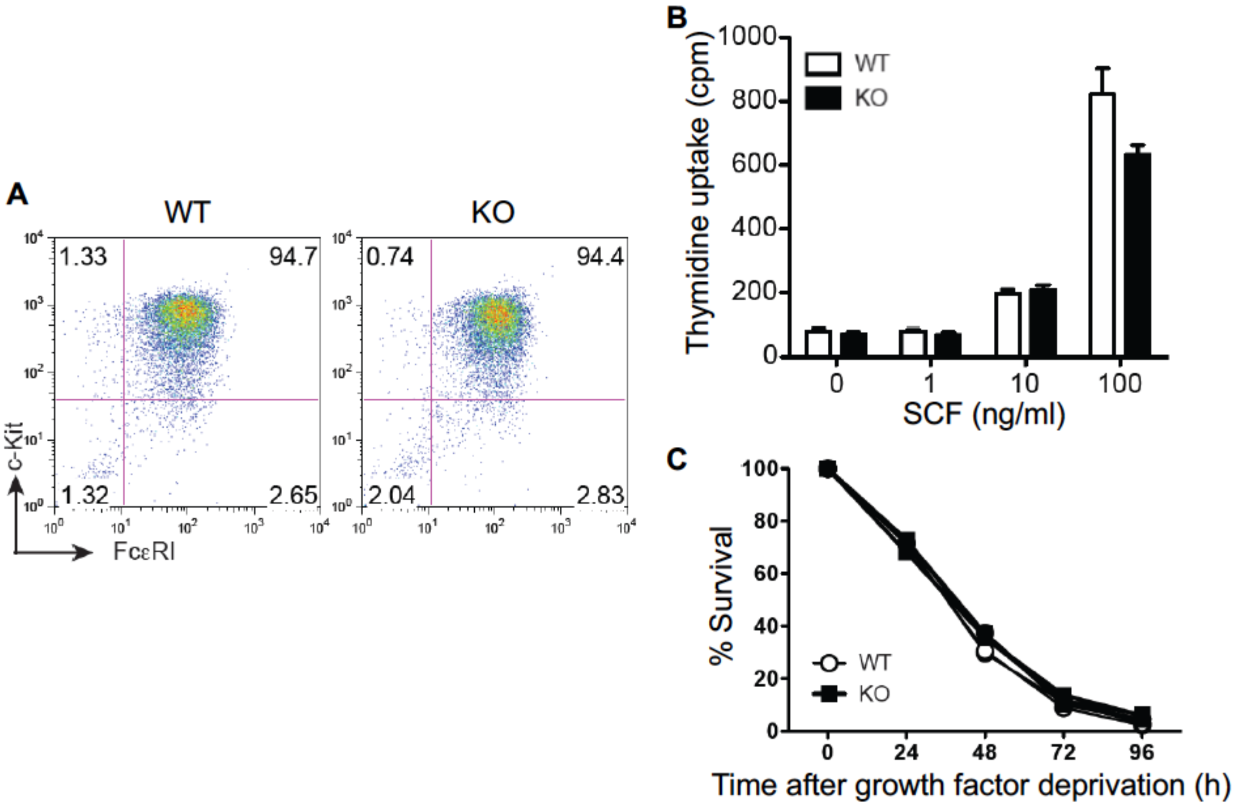

Figure S1. Mast cell differentiation, proliferation, and growth factor deprivation-induced death are not affected by MAVS deficiency. Bone marrow cells from femurs from WT and *Mavs*$^{-/-}$ mice were cultured in IL-3-containing medium for 6 weeks. (**A**) Mast cell differentiation was analyzed by expression of FcεRI and c-Kit by flow cytometry. (**B**) Proliferative activity was measured by [^{3}H]thymidine uptake. (**C**) Growth factor (i.e., IL-3) deprivation induced cell deaths by apoptosis.

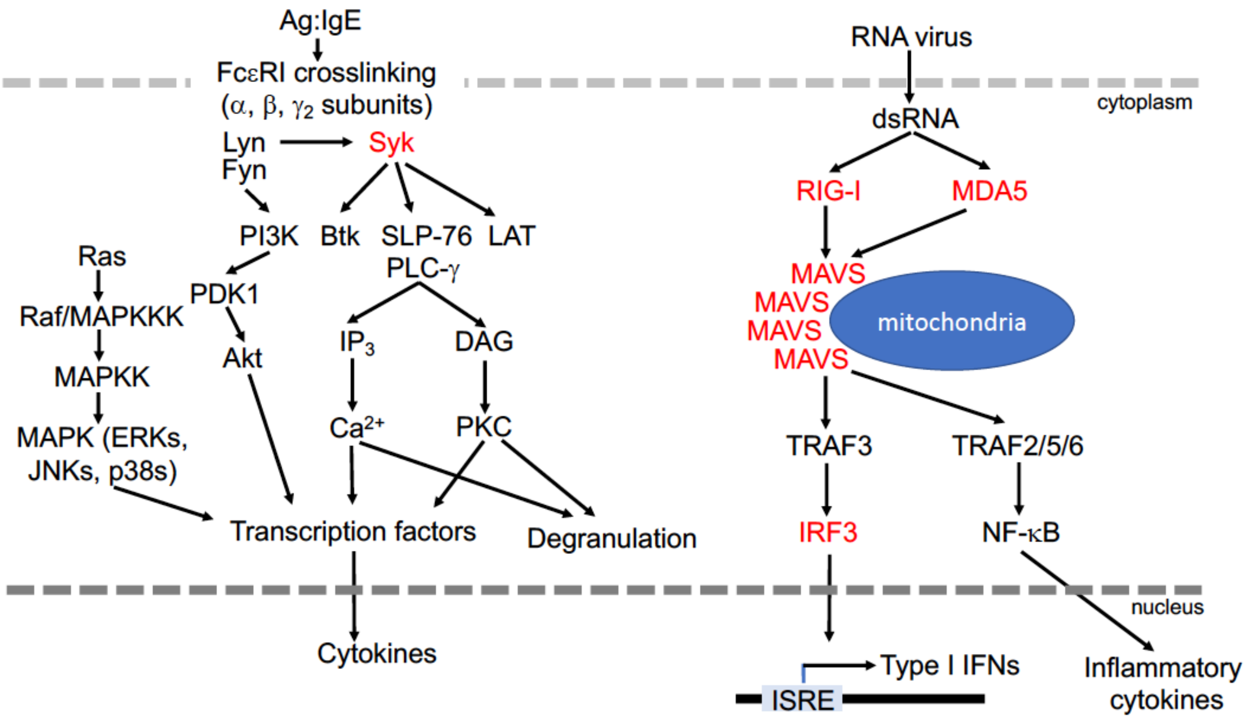

Figure S2. FcεRI and antiviral signaling pathways relevant to this study. FcεRI signaling pathway (Left) and antiviral RLR signaling pathway (Right) are shown with main signaling molecules and related functions. dsRNA, double-stranded RNA; ISRE, interferon-sensitive response element.
